# Variation in the Helical Structure of Native Collagen

**DOI:** 10.1371/journal.pone.0089519

**Published:** 2014-02-24

**Authors:** Joseph P. R. O. Orgel, Anton V. Persikov, Olga Antipova

**Affiliations:** 1 Departments of Biology, Physics and Biomedical Engineering, Illinois Institute of Technology, Chicago, Illinois, United States of America; 2 Pritzker Institute of Biomedical Science and Engineering, Illinois Institute of Technology, Chicago, Illinois, United States of America; 3 BioCAT, Advanced Photon Source, Argonne National Laboratory, Lemont, Illinois, United States of America; 4 Lewis-Sigler Institute for Integrative Genomics, Princeton University, Princeton, New Jersey, United States of America; Massachusetts Institute of Technology, United States of America

## Abstract

The structure of collagen has been a matter of curiosity, investigation, and debate for the better part of a century. There has been a particularly productive period recently, during which much progress has been made in better describing all aspects of collagen structure. However, there remain some questions regarding its helical symmetry and its persistence within the triple-helix. Previous considerations of this symmetry have sometimes confused the picture by not fully recognizing that collagen structure is a highly complex and large hierarchical entity, and this affects and is effected by the super-coiled molecules that make it. Nevertheless, the symmetry question is not trite, but of some significance as it relates to extracellular matrix organization and cellular integration. The correlation between helical structure in the context of the molecular packing arrangement determines which parts of the amino acid sequence of the collagen fibril are buried or accessible to the extracellular matrix or the cell. In this study, we concentrate primarily on the triple-helical structure of fibrillar collagens I and II, the two most predominant types. By comparing X-ray diffraction data collected from type I and type II containing tissues, we point to evidence for a range of triple-helical symmetries being extant in the molecules native environment. The possible significance of helical instability, local helix dissociation and molecular packing of the triple-helices is discussed in the context of collagen's supramolecular organization, all of which must affect the symmetry of the collagen triple-helix.

## Introduction

**Figure 1:**
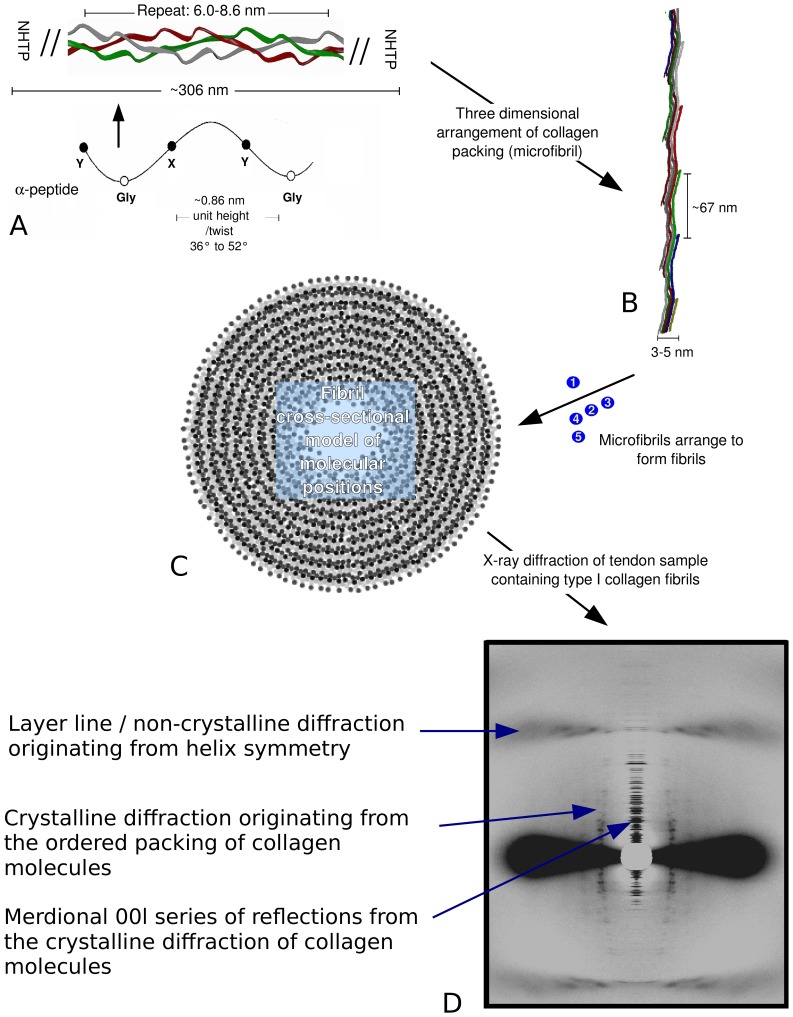
Greatly simplified organizational hierarchy of fibrillar collagen structure (from polypeptide to fibril) A. The collagen-forming polypeptide chains contain a large helix-forming domain with the repeat amino acid sequence Gly-X-Y, where X and Y are occupied by Pro or Hyp more frequently than other residues, but only account for approximately 1/6 of the total amino acid content (see for instance human sequence: ExPASy sequence data bank codes; P02452 and P08123). An arrow points to the figure element that shows that three polypeptides form the collagen monomer. The large triple-helix (super-helix) domain of approximately 300 nm in length is flanked by non-helical telopeptides (N and C, shown). The 6–8.6 nm dimension indicates the repeat of the triple-helix (36; 37). B. Collagen molecules are staggered approximately 67 nm from one another in the formation of microfibril aggregates. The microfibrils are D-periodic (D = 67 nm), and in each D-period, two monomers coil, or partially coil, around each other giving the appearance of another helix-like feature in the structural hierarchy (3). C. Cross-sectional view of the collagen molecular packing of a type I collagen fibril (11). Each circle represents one collagen molecule in cross-section (at the axial level of 0.44D). at the 0.44 D position. Next to B to C arrow, cross-section of an isolated microfibril. D) Archival image (Orgel laboratory) of the wide angle fiber diffraction pattern of type I collagen from rat tail tendon. The distinctly different but superimposed non-crystalline and crystalline diffraction patterns are indicated. Previous fiber diffraction studies of collagen's helical structure have concentrated on the non-crystalline part of the pattern, in this present study, we analyze crystalline diffraction data.

The structure of fibrillar collagen is complex and multifaceted (Figure 1) [1], [2]. In general, most structural studies of collagen have focused on either its triple-helical composition or its fibrillar arrangements. However, collagen's molecular packing is also key in understanding its overall structure and function [1]–[3]. This packing structure is directly effected by and affects both collagen's helical and fibrillar organization [4], (literally) providing the framework to understand otherwise context-less stretches of peptide sequences that cells recognize and interact with [1], [2], [5], [6].

X-ray fiber diffraction has been a critical tool in elucidating the structure of the molecular packing arrangement, which in turn, has allowed insights into other aspects of the internal molecular organization [3]. The highly crystalline packing of collagen molecules in the direction of the collagen helix in some tissues in particular, allows the collection of crystalline diffraction patterns [7]. Unlike many fiber diffraction patterns which show only the unsampled molecular transform of the sample (non-crystalline diffraction arising from the helical symmetry), these patterns also contain Bragg peaks originating from the well ordered axial and lateral packing of collagen molecules into fibrils (Figure 1D). This crystalline diffraction from collagen tissues may be treated as analogous to that from a single macromolecular crystal. Except that it arises from many fibrils within the sample, giving rise to cylindrical and rotational averaging effects and the inherent complications that its multi-crystallite composition imposes (such as overlapping Bragg peaks and in-coherent scatter in the off-meridional regions) [4], [8], [9]. The meridional section of the diffraction pattern does not suffer from these issues however. Furthermore, it extends to medium angle resolution and demonstrates a potentially very high degree of order within the axial packing arrangement which is in the same orientation as the helix [4], [10].

Such crystalline diffraction data, arising from the axial and lateral packing, has been used to determine the molecular packing structure of type I collagen *in situ* [4]. In this previous work, both the native and 2Fo-Fc electron density maps, constructed from experimentally determined phases and observed amplitudes, showed good agreement. The observed diffraction and the simulated pattern calculated from the model fitted to the experimental electron density map also showed good agreement. In an effort to develop the most accurate model possible from the available data, coordinates composed from high-resolution collagen-like peptide structural data [11] were fitted into the low-resolution electron density map, which is essentially a molecular envelope. This approach is analogous to that commonly used with cryo-electron microscopy or SAXS data whereby higher-resolution structural data are “docked” into the molecular envelopes defined by the low-resolution data, [12], [13]. As such, it would be inappropriate to expect the structural models from such studies to exactly match that derived from high-resolution single-crystal crystallography or multidimensional NMR of molecular model peptides. In the same vein of thought, however, since they are experimentally determined and not solely *in silico* models they are of significant value if used appropriately (and benefit from the same highly advanced force field calculations used in pure modeling studies [14], [15] while retaining explicit experimental data verification).

In the present study we consider what diffraction data arising from the molecular packing structure of collagen can say regarding the helical organization of the fibrillar collagen's. Our objective is not to prove that one particular helical symmetry is prevalent, but to demonstrate that there appears to be multiple conformations in fibrillar collagen's helical domain that include variations other then the dominant symmetry question. This possibility has been predicted from short model peptides of collagen or collagen-like sequences but has not yet been shown for the full molecule in its natural context [16], [17]. In connection to the symmetry of the helix, our approach avoids potential pitfalls in model fitting to ‘less-than-perfect’ helical diffraction by primarily using the Patterson function to detect helical periods in the crystalline meridional data of two different fibrillar collagen systems. Further observations are made via the use of experimentally determined models and ‘ideal’ helical models composed to fit expected helical symmetries described in the literature [11], [16], [17]. Finally, since the triple-helix of type I collagen deviates from the expected triple-helix structure in ways that are not merely symmetry related [3], [16], [18], [19], we calculated sequence based predictions of local triple-helix stability to see if these features are only stability based or if other factors (such as lateral packing and bends in the molecules) might play a role in defining collagen's helical structure. If these variations, such as helix ‘puckering’, always correlate to low thermal stability regions then it could be assumed that lateral packing and other molecular interactions with collagen have little effect on its helical structure.

Using meridional data from type I and type II collagen containing tissues we have detected periodic functions in the native Patterson function that may indicate the underlying helical symmetries of both these fibrillar collagens. Together with structural data obtained from an earlier three dimensional packing structure of type I collagen [3], [4], this provides insight into variations in the helical organization of the collagen triple-helix within the fibrillar collagen's ‘helical domain’.

## Materials and Methods

### Fiber Diffraction and Coordinate Data

X-ray fiber diffraction data from native, hydrated, rat tail tendon and lamprey notochord were obtained in previous studies [4], [10], [20]–[22]. The scaled amplitudes of the central, meridional section of each data set were used to calculate Patterson functions, whilst the contributed coordinates from Orgel et al., 2006 [4] were used to for displaying aspects of collagen's molecular and packing structure (Figure 1).

The type II data is published in supplementary information of Antipova and Orgel [22], the type I data is to be found via the linked RCSB codes (3HR2 and/or 3HQV).

### PDB Models

Structure factors were calculated for each model to generate model based structure factors for comparison with those determined experimentally. These were used in the Patterson Function calculations (below).

The coordinates of an experimentally determined type I collagen model structure (RCSB ID: 3HR2) (3; 14) that contains the fibrillar conformation of the collagen molecule (including its packing) were used for comparison with native collagen.

The crystal structure of the (PPG)_10_ triple-helical peptide from crystals grown in a microgravity environment with an “ideal” 7/2 symmetry (RCSB ID: 1K6F [23]) was used to simulate an “ideal” full-length collagen with perfect 7/2 helical twist. The 3rd position prolines were substituted to the Hyp (4-R-hydroxyproline) residues and the structure was translated into full-collagen length with (Gly-Pro-Hyp)_338_ sequence.

All available high-resolution crystal structures at the time this research was conducted, were used to get as close as possible to the idealized triple-helix with 10/3 symmetry [11] and the portion of type III collagen peptide in particular (PDB ID: 3DMW [17]) was used as a pattern with an “ideal” 10/3 helical twist. This included residues 6–20 of chain A, residues 6–17 of chain B, and residues 6–17 of chain C of 3DMW, with 33 residues in total, making one triplet overlap for a complete period. This structural unit was mutated into (Ala-Ala-Gly) repeating structure (with zero imino acid content to disfavor the 7/2 helix). To adjust positions of all atoms, the idealized alpha-carbons were fixed and the rest of the structure was energy minimized using AMBER forcefield [24]. Finally, this idealized structure was translated to give a full-length (helical portion) collagen molecule with (Ala-Ala-Gly)_338_ sequence and the ideal 10/3 helical twist.

Each structure was superimposed (without changing its internal structure) on the native type I collagen molecule to give the same degree of relative tilt to the unit cell [9]. Two more models were composed from the GAA and GPO coordinates by substituting the type I collagen sequence onto each structure coordinate atoms (GAA colseq and GPO colseq respectively). The side chains of the amino acid sequence of type I collagen (that seen in 3HR2) replaced the corresponding X and Y-positions, glycine positions were unaltered. The structure factors were calculated for each model so that Patterson functions could be made.

### Patterson Function

The one dimensional Patterson function for the 00l (meridional diffraction series) was calculated:
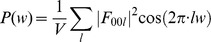



Where 00l are the Miller indices of the (one dimensional) unit cell, |F_00l_| the amplitude and w is a point in unit cell space. The significance of the function is that the peaks in a Patterson function refer to the distances between *repeating* electron dense regions (such as helically organized electron densities) within the crystalline unit cell of the diffraction sample. The series terms are the scaled square amplitudes [4], [10], [22]. In short, the Paterson function is a pair correlation function for electron density, the peaks of which reveal periodicities in interatomic distances.

### Triple Helical Stability

The triple-helical stability was calculated via the “Collagen Stability Calculator” [25]: http://compbio.cs.princeton.edu/csc/profile.html, for the rat sequence helical domain (uniprot P02454, so as to match the type I experimental data). The plot was inverted so that increasing instability gave peaks rather than troughs. This allowed the results to be compared straightforwardly with a plot of helix dissociation. The latter was calculated from the average Calpha deviation of the ‘relaxed’ model (3HR2) from the ‘rigid’ model (3HQV) of the *in situ* collagen helix, after the method of Perumal et al [3].

## Results and Discussion

**Figure 2:**
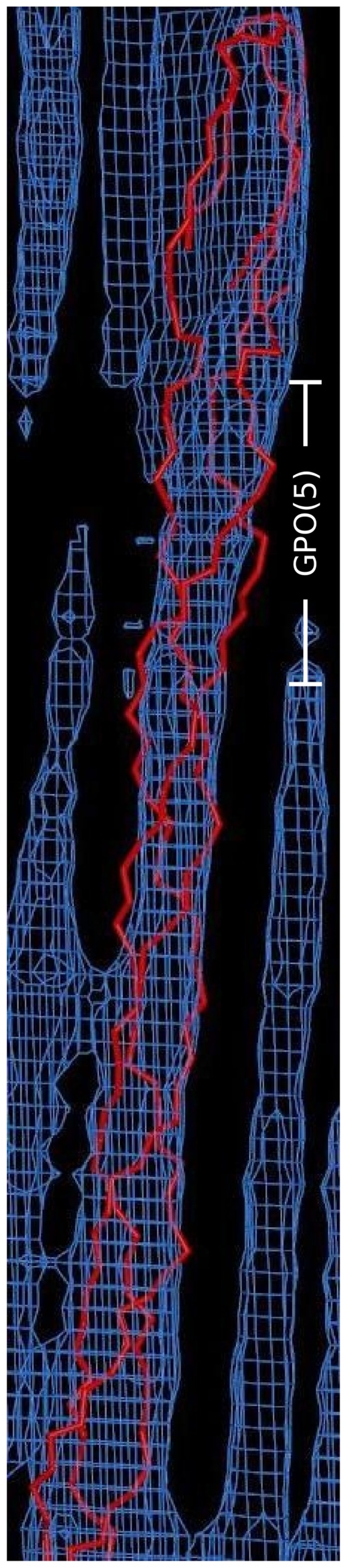
Helical and non-helical organization of collagen. The non-helical, folded C-terminal end of the collagen molecule (top) extending from the triple-helical region (below). The electron density of neighboring collagen molecules can be seen along side the chain traced segment (red). The GPO_5_ domain is indicated in white.

**Figure 3:**
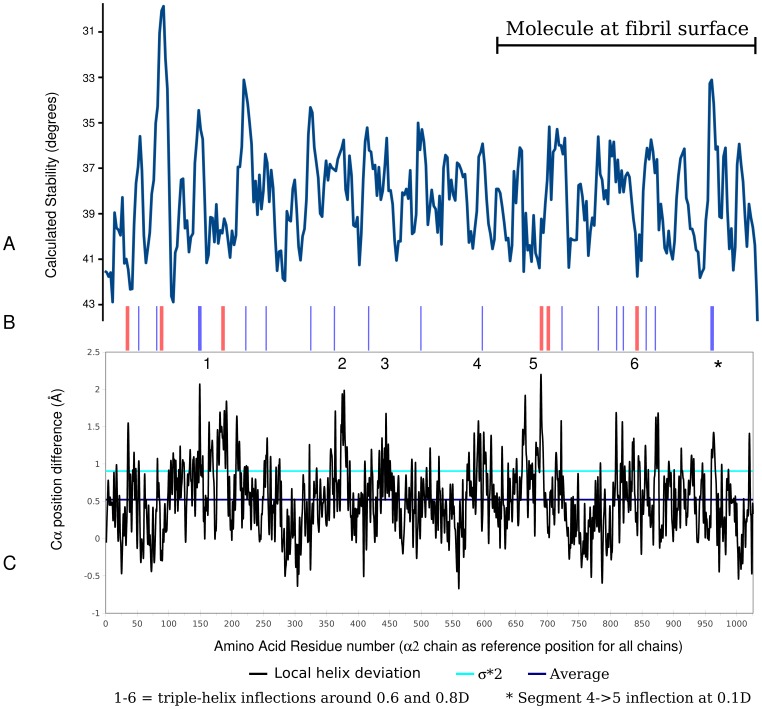
Thermal stability versus α chain triple-helical dissociation. A) Thermal stability plot [25]. B) Comparison of local predicted stability variations with local helical dissociation. Blue lines mark a noteworthy correlation between peaks that indicate thermal instability of the helix and where the helix is also dissociated (see part C). Red lines indicate noteworthy areas where there is not a correlation. The helix is calculated to be thermally stable but the low resolution structural data indicates the triple-helix to be relatively dissociated at room temperature. Or vice versa, the stability plot indicates a well formed helix while the structural data shows a relatively disassociated one. Some of the places were there is no correlation (stable helix but structure shows a dissociated one) are located at points of molecular inflection (bends, see Figure 1 and 3 and electron density [4]). Thicker lines indicate more significant discrepancy/correlation, unmarked areas are thought to show more or less expected similarity between the two plots (A and C). C) dissociation of peptide chains: difference between the calculated relaxed model (via force field calculations against diffraction data) and the starting stringent model (from high resolution model peptide data) of the collagen triple-helix. Sequence numbering includes the N-telopeptide residues. This is an estimate of triple-helix dissociation. The magnitude of dissociation of the three peptide chains are shown as a local average (black line), along with the global average (blue line) and two times standard deviation (2*σ, light blue line). 1–6 and * indicate significant bends in the collagen molecule as determined from the electron density of *in situ* fibrillar collagen data.

Previous analysis of the model and electron density of the type I collagen *in situ* packing structure [4], [10], [26], [27] showed that the fibrillar collagen molecule is composed of triple-helical and non-helical domains (Figures 1 and 2). The lateral packing of neighboring collagen molecules is quasi-hexagonal, the intermolecular cross-links that help maintain this relationship being known to be formed via the non-helical telopeptides. The (GPO)_5_ domain directly proceeding the C-terminal telopeptide (Figure 2) is the collagen sequence most closely related to studies of collagen-like peptides such as (GPO)_10_ [28]. The electron density of this region indicates a well-formed triple-helix for most of this domain, except for the last 2 repeats where it connects to the non-helical telopeptide. Here the more bulbous electron density expands beyond the normal diameter of the triple-helix as it describes the outline of the folded α1 chains. This indicates the triple-helix begins to associate at the (GPO)_5_ region as suggested previously [29], [30], but must transition from the non-helical conformation to the triple-helical conformation over one or more GPO repeats. Other sections of the ‘triple-helical domain’ are similarly not entirely triple-helical. For instance, the α chains are more dissociated from the center of the triple-helix than data from high-resolution, model peptides, can detect due to their short length and required sequence bias (high imino acid content) [3], Figure 3.

### Helical instability versus helix dissociation

Is helix dissociation, a significant deviation of helix structure from the ideal (and therefore also perfect symmetries), a persistent structure or due to helical instability?

Several short regions of the fibrillar packing structures [4] helical domain are bent (and there is a significant change in direction of the molecular helix before and after the bend) and have a larger diameter then the rest of the density representing the triple-helices. The triple-helical models (3HR and 3HQV) fitted to these electron densities accommodate these bends by bulging outward (a triple-helix local disassociation) presumably also to accommodate the electron-density which has a larger diameter then the average helix in these locations. In a subsequent investigation [3], it was found that the α chains are significantly disassociated at several other sites (Figure 3). The points at which the helix is most disassociated are at those points of molecular inflection (as indicated, Figure 3). That is, where the molecular segments bend within each D-period to accommodate their re-arrangement as they progress into the next D-period (see microfibril in Figure 1).

Local disassociation of the triple-helix appears to coincide with *both* regions of calculated relative instability and stability. In Figure 3, regions 1-6 correspond to the bends that accommodate molecular segment re-arrangement. Regions 2–4 show a correlation between relative peaks in thermal instability and triple-helix dissociation. Regions 1, 5 and 6 show regions of relative helical stability with relatively large triple-helix dissociation. The latter case is consistent with the proposal that deviation from the helical ideal is not an artifact from thermal fluctuation, but a persistent structure (see red lines, Figure 3). This assertion is supported also from the molecular packing structures electron density [4], or otherwise those thicker sections of electron density where the helix bends, would not be visible to X-ray diffraction. I.e, those bends must persist over millions of crystallites (fibrils) within the tissue sample.

In general, these and other areas of significant dissociation correlate well with the calculated local stability of the triple-helix (Figure 3A) albeit with some evident differences. Some areas that show helix dissociation where the thermal calculation proposed an increased stability, and vice versa (non-dissociation in regions of proposed low stability) are indicated in Figure 3B with red lines. This latter point does not indicate a discord, but perhaps shows that at least some of these dissociated areas are disassociated due to the bend of the helix but are otherwise stabilized by the closely spaced neighboring helices. This possibility is supported by the fact that such areas appear to cluster around the inflection points of the molecule (marked 1–6 and *, Figure 3). However, the majority of the disassociated triple-helical areas correlate well with the low thermal stability regions, indicating that local amino acid sequences generally determine fluctuations in triple-helix stability and local helix dissociation.

When taken into account along-side the periodicities found in the X-ray diffraction terms from type I and II collagen showing intermediate structures to the 10/3 and 7/2 helices (see below), these data also indicate that collagen's helical structure is more diverse than that observed in short relatively uncomplicated model peptides. This should not be too surprising as there are factors not easily accounted for in most studies of collagen helix structure or stability: most of the triple-helix is closely packed next to neighboring triple-helices and at other times are closely associated with ECM ligand binding proteins at the fibril surface [6]. The potential helix stabilizing effect of inter-molecular packing may explain instances were the calculated stability indicates an unstable helix, while the structural data indicates the helix is well formed (red lines, Figure 3 B). This is seen particularly for the very low thermal stability region between 75 and 100 that with a small exemption, shows a well formed helix despite low predicted stability. Regions in Figure 3C marked 1, 5 and 6 also show similar discrepancies, but all correspond to regions of helix bending that occur in a region of the D-period (the gap region) that is heavily substantiated with the proteoglycan decorin [31], [32] that could contribute to stabilizing those corresponding regions of the triple-helix.

### Variations in helical symmetry

**Figure 4:**
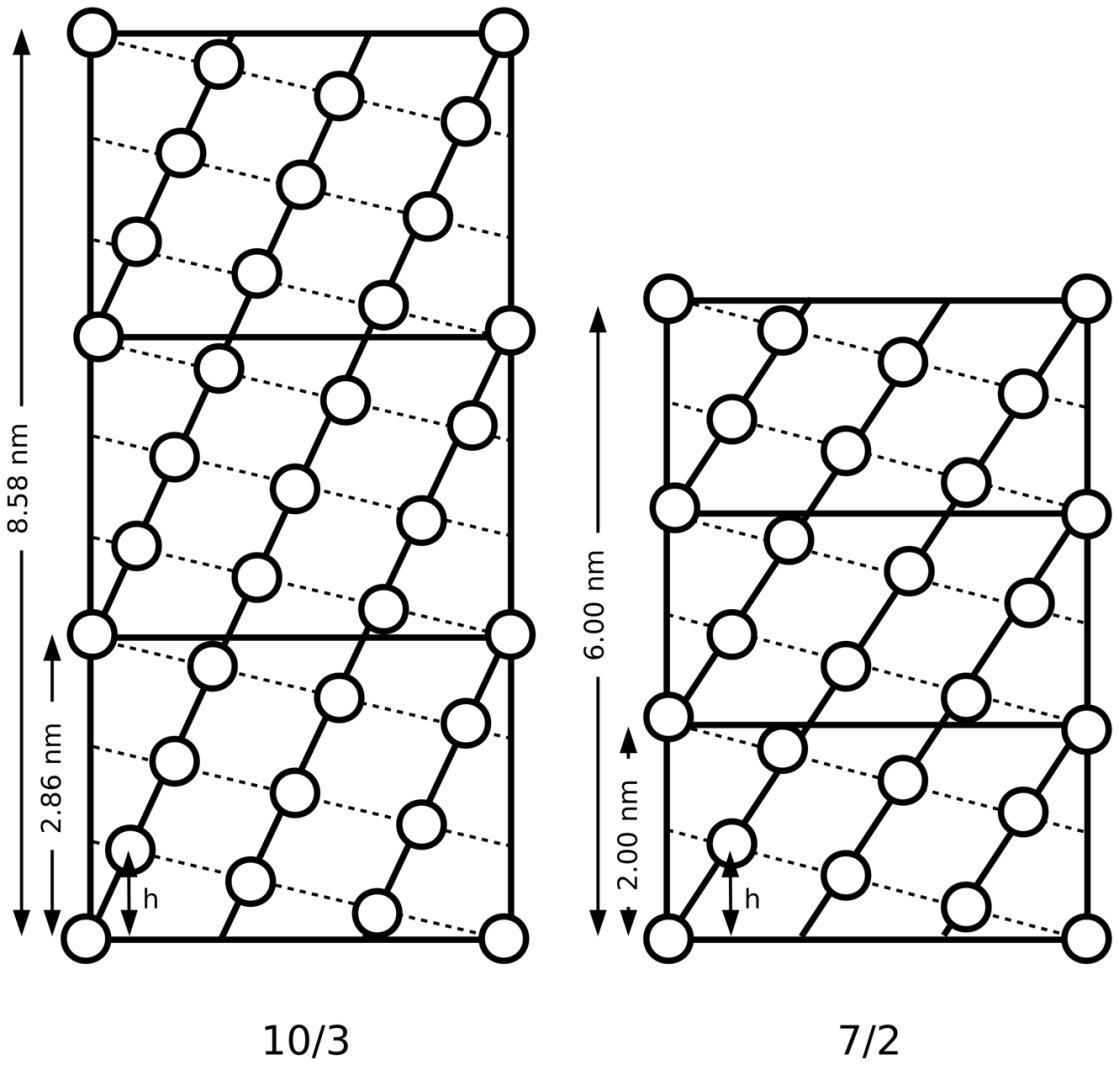
Helix net map of the 10/3 (A) and 7/2 (B) triple-helix models. The unit height of the α-peptide chain (h) and the superhelix (h_sh_), the pitch and helix true repeat periods of each helical symmetry is as indicated.

There are currently two well described symmetries for the collagen triple-helix: the Rich and Crick/Fraser et al model describing the triple-helix and a 10/3 helical symmetry for it [33], [34], and the Cohen and Bear/Okuyama et al., [28], [35], [36] 7/2 helix symmetry structure. The average conformation of both is a left-handed triple-helix with either 10 repeating GXY triplets in 3 turns (10/3) or 7 repeating GXY units in 2 turns (7/2). Each possess unique periodic values, a pitch of 2.86 or 2 nm for the 10/3 and 7/2 respectively, and a ‘true repeat’ [37] of 8.58 and 6 nm for 10/3 and 7/2 respectively (Figure 4). There has been a considerable amount of discussion regarding which of these structures represents ‘real’ collagen structure, with investigations of amino acid and imino acid rich short collagen-like peptides and re-analysis of their data [11], [38]–[41] to find evidence of 10/3 or 7/2 helical twists [38]. Others re-investigated the molecular transform fiber diffraction data to examine the same question [34], [42]. Each of these studies has its inherent strengths and weaknesses. The collagen-like short peptide studies offer high-resolution structures and suggest that high imino acid content leads to helices that are closer to 7/2 symmetry, while the peptides with low imino acid content and lower stability tend to have a helical twist (but not necessarily the expected period repeats) closer to the 10/3 (26). The fiber diffraction studies of the molecular transform, have relied on discrete model based studies, which introduces the concern of non-unique solutions or that they may overlook that a possible *range* of conformations exist [42]. In addition, the fact that early studies of the helical conformation relied on highly stretched samples ∼10% [34] leads to the possibility that the triple-helix itself may also be stretched (in this case in favor of the 10/3 helix). Other studies may find alternate conformations from diffraction data collected under conditions that may not be the same. Even a fiber diffraction study that did not suffer from these model ambiguities and made use of taut but not highly stretched tendons [4] could not (directly) address the question of helical symmetry properly due to its anisotropic resolution. However, the crystalline diffraction data from this [4], [10] and related studies [22] may be used to look for evidence of helical periodicities associated with one helix form or another without *a priori* model bias.

**Figure 5:**
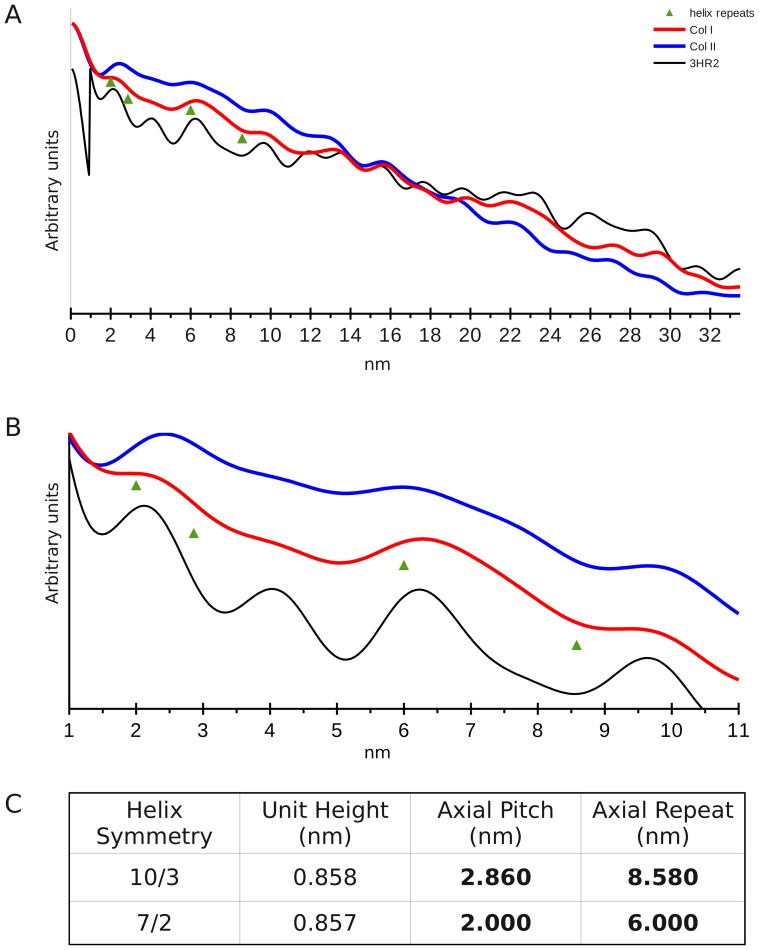
Patterson functions of the type I and II collagen 00L (meridional) series. A) Patterson function from 0.0–0.5D, the inverse (0.5–1.0) half of the Patterson function is not shown. The fractional distances between periodicities indicated in the functions has been multiplied by 67 nm (the length of the one dimensional unit cell – the D-period) for comparison with the helix symmetry periods. B) Enhanced view of the Patterson function range of interest for the helix symmetry periodicities. C) Table of key helix periodicities for comparison with A and B (see also Figure 4).

**Figure 6:**
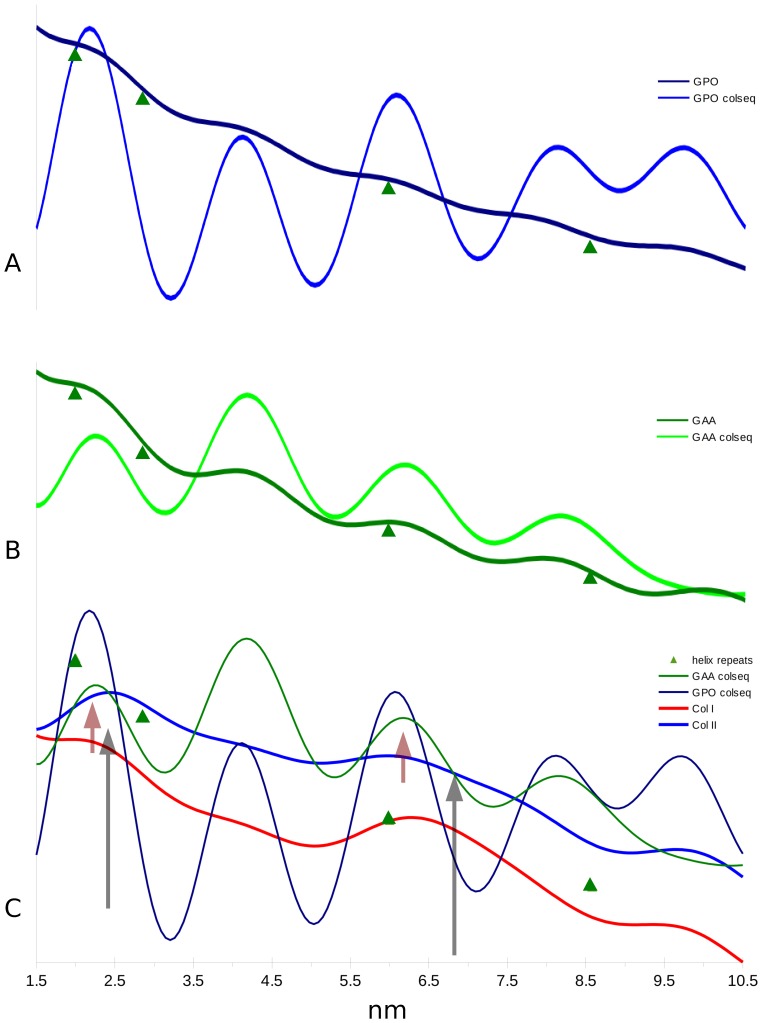
Patterson functions of collagen model structure factors 00L (meridional) series. A) Comparison of GPO (7/2 model) and GPO with collagen sequence threaded to check if amino acid sequence effects periodicities detected by the Patterson function. It does not appear so. B) As (A) except for GAA (10/3 model). C) Patterson functions of collagen types I and II are compared with those from the GAA and GPO coordinate models with the collagen sequence threaded onto them. The semi-transparent arrows mark: red, the maximum of the GAA (10/3) helix model pitch and repeat periods, the black arrows mark the collagen I and II respective positions for these periods. Note that the collagen experimental data show periods that are longer then the 7/2 and do seem to almost reach the 10/3 expected range. This could be interpreted to mean that both helical symmetries are found in native fibrillar collagen in addition to other possible conformations.

The native Patterson function plots of meridional data (relating to the axial, including helical, structure) from rat tail tendon (collagen type I) and lamprey notochord (collagen type II) show several common features (Figure 5). Of interest to this study is the range of periodicities detected between 0 and 11 nm (Figure 5B). Although the unit height of both the 10/3 and 7/2 helical models at ∼0.86 nm (Figures 5 and 6) is below the detectable threshold of the plots (the ‘self’ period of the Patterson map is steep and uninterpretable for this region), periodicities above 1.5 nm for type I and 1.9 nm for type II are clearly seen as peaks for both data sets (Figure 5). Since the type I dataset has a resolution of ∼0.5 nm and the type II 1.9 nm, periodicities detected above these respective values are within the probable competence of the data. Periodicities that may correspond to the helical pitch and true repeat are clearly seen (Figures 5 and 6) and are recorded in Table 1 (along with the other periodicities observed).

**Table 1:** Patterson function periodicities and correlation between observed and ‘perfect’ helical symmetry periodicities (see also Figure 5).

Patterson axial peaks	Col I (nm)	Col II (nm)	Average (nm)	Observed Repeat	Observed Pitch Ratio	7/2 Pitch Ratio	10/3 Pitch Ratio	Expected Pitch Ratio
1 (p)	02.40	02.45	02.43		(p = 2.43)	(p = 2.0)	(p = 2.86)	
2 (a)	06.40	06.20	06.30	Repeat a (7/2?)	2.60	**3.125**	2.19	3
3 (b)	7.70*	07.70	07.70	Repeat b (10/3?)	**3.18**	3.85	2.69	3
4 (c)	09.80	09.90	09.85	Repeat c (10/3?)	4.06	4.94	3.45	3
5	13.40	13.00	13.20			Average 3&4	**3.07**	3
6	15.80	15.70	15.75					
7	17.80	-	17.80	The ‘ideal’ helix symmetry for either 7/2 or 10/3 is not observed but related periods are. Further, it appears that there may be at least one other intermediate (or spectrum) of helical symmetries in ‘real collagen’ as both pitch and repeat periodicity peaks are broad (see Figures 5 and 6). (p) is pitch. (a) – (c) are observed candidate helix repeat distances.				
8	19.80	19.50	19.65					
9	22.10	22.40	22.25					
10	23.90	25.00	24.45					
11	27.30	27.10	27.20					
12	29.50	29.40	29.45	*Peak is shallow				
13	31.70	31.70	31.70					

A pitch with a true repeat ratio of 3∶1, a non-integral helix (an integral helix being one were the true-repeat and pitch are the same, such as in B-form DNA), would indicate that the pitch and repeat values are well paired (8.58/2.86 = 6.0/2.0 = 3.0). The observed, average periodicity that appears to correspond to an average pitch value of 2.43 nm (from an admittedly broad range) provides a product of 2.6, 3.18 or 4.06 when possible repeat values of 6.3, 7.7 and 9.85 nm are used (observed values in the Patterson functions). Both the 2.43 nm pitch and 7.7 nm repeat values being intermediate between the 10/3 and 7/2 helical period values. However, the existence of three distinct period values that may correspond to helical repeats (the 6.3, 7.7 and 9.85 nm values) could indicate at least three distinct helical symmetries in collagen samples with minimal stretch. The fact that the 2.43 nm ‘pitch’ periodicity is broad, could also indicate the existence of both the 10/3 2.86 nm and 7/2 2.0 nm pitches. As, Table 1 indicates, that the average of the 7.7 and 9.85 nm ‘repeat’ periodicities is very close to the ‘perfect’ 10/3 helix repeat whilst neither individual value is appropriate. The same is true of the 7/2 helix repeat value, 6.3 nm being greater than that expected.

Given the strong showing of periodicities that are in the range of the helical symmetry periods expected, it would be straightforward to envision regions of the collagen molecules that conform to the ideal 10/3 or 7/2 helical symmetry. The values seen in the Patterson maps (in Figures 5 and 6) correspond to averages of structures found within the samples, even though they do appear to cluster around three observed repeat values. The ideal 10/3 structure periodicity is not observed, but two values (7.7 and 9.85 nm) may represent evidence for the existence of related symmetries whose average is 10/3. Neither is the ideal 7/2 structural periodicity observed, although the repeat value of 6.3 nm is closer to the expected ideal than its corresponding 10/3 structure. A caveat to be considered here, is that stretching for at least tendon samples does in fact represent something not unlike its native state. It is quite possible that the work of Fraser et al [34] represents an accurate definition of 10/3 structure in tendon samples, if that stretching causes the normalization of the molecules helical symmetry towards the larger repeat values. That the type I and type II collagen samples produced similar, but not identical values may also indicate variation in the molecular conformation of the helix as per its tissue context. It is also a possible consequence of the type II collagen notochord samples being stretched, perhaps to a greater extant then the type I tendon samples to aid in the recording of meridional diffraction over that of lateral packing diffraction from a second population of fibers [22]. This could also explain why the type II Patterson functions give indications that are apparently closer to the ideal 10/3 parameters then that from the type I data used in this study [4], [10], Figures 5 and 6.

Although Fraser et al [34] and Okuyama et al [42] when comparing *a priori* helix models against molecular transform fiber diffraction data, utilized occupancies as low as 30% for imino acid atoms rather then contend with the amino acid sequence data (or lack of it in Fraser et al's case), both studies showed a deeply significant fact: They (when taken together) successfully demonstrated that even imino acid rich sequences in the collagen molecule may adopt more than one type of helical symmetry (Figures 2, 5 and 6). It is further supported by other studies which showed variations in helical symmetry between imino acid rich and imino acid poor regions [11]). Although neither symmetry (7/2 or 10/3) are ideally represented in either the medium resolution structure of Orgel et al 2006 [4], or the periodicities seen in the native and model Patterson function of the 00L (meridonal) series of this study for both types I and II collagen (Figure 5), it would seem that there is evidence for the presence of closely related structures to both symmetries in molecular collagen *in situ*.

A possible concern that the Patterson function could be detecting common sequence repeats rather then primarily the axially repeating helical periods (Table 1, Figures 5 and 6) is mitigated by data presented in Figure 6. The same periodicities of interest, between 2 and 8.6 nm, are detected unchanged between the GAA/GPO models and their corresponding coordinates onto which the type I collagen sequence was substituted. That is, the periodicities of the collagen sequence do not change the positions of periodicities seen between the different helix models. If anything, the magnitude of the signals that seem to correspond to helical symmetry periods are enhanced. Furthermore, it can be seen that even these models based on structures derived from high-resolution single crystal studies with the perfect helical twists, indicate a helical period range that is not the ideal 7/2 and 10/3 structures determined from fiber diffraction studies on full length collagen. Perhaps their short length and more likely, the absence of the long scale molecular packing interactions seen in fibrillar collagen effect this aspect of helical structure. However, they do provide us with simple, useful insight into this analysis. The GAA model periodicities show a clear increase in period over the GPO model of around 0.12 nm. A comparison between the helical model data with the experimentally obtained data for types I and II collagen indicate that the native collagens have periodicities that are more similar to the GAA (10/3) helix model then the GPO (7/2) model in the placement of the pitch and repeat distance parameters (Figures 5c, 6c and Table 1) [33], [34]. We suspect that both helical symmetries along with other more poorly defined ones (see helix instability above and Figures 3, 5 and 6) may be found within the fibrillar collagen triple-helical domain. If so, this may help in explaining the diffuse, non-crystalline nature of the helical diffraction part of the collagen fiber pattern, as it would represent an average of these symmetries. The fact that the high-angle layer line index of the 10/3 and 7/2 helix accounts for the same layer-lines seen in the non-crystalline diffraction [42] may not be an accident, but representative of the persistence of both symmetries in the same sample, especially when stretched or not, allowing a transformation to occur from one symmetry to another [43].

There is much yet to be resolved concerning collagen structure, even with the great strides in progress made recently by the various groups referred to in this study. We anticipate that future work will need to increase its reliance on the use of composite data from collagen-like peptides incorporated into the context of lower resolution structural determinations from techniques such as cryo-electron microscopy and fiber diffraction to garner the better, most representative picture of collagen nature.
